# Evaluation of older persons’ medications: a critical incident technique study exploring healthcare professionals’ experiences and actions

**DOI:** 10.1186/s12913-021-06518-w

**Published:** 2021-06-07

**Authors:** Malin Holmqvist, Johan Thor, Axel Ros, Linda Johansson

**Affiliations:** 1 Department of Hospital Pharmacy, Region Jönköping County, and Department of Biomedical and Clinical Sciences, Linköping University, Linköping, Sweden; 2 The School of Health and Welfare, Jönköping University, Jönköping, Sweden; 3 Jönköping Academy for Improvement of Health and Welfare, the School of Health and Welfare, Jönköping University, Jönköping, Sweden; 4 Futurum, Region Jönköping County, and Department of Biomedical and Clinical Sciences, Linköping University, Linköping, Sweden; 5 Institute of Gerontology, Aging Research Network-Jönköping, the School of Health and Welfare, Jönköping University, Jönköping, Sweden

**Keywords:** Patient safety, Polypharmacy, Older persons, Healthcare professionals, Critical incident technique, Medication evaluation

## Abstract

**Background:**

Older persons with polypharmacy are at increased risk of harm from medications. Therefore, it is important that physicians and nurses, together with the persons, evaluate medications to avoid hazardous polypharmacy. It remains unclear how healthcare professionals experience such evaluations. This study aimed to explore physicians’ and nurses’ experiences from evaluations of older persons’ medications, and their related actions to manage concerns related to the evaluations.

**Method:**

Individual interview data from 29 physicians and nurses were collected and analysed according to the critical incident technique.

**Results:**

The medication evaluation for older persons was influenced by the working conditions (e.g. healthcare professionals’ clinical knowledge, experiences, and situational conditions) and working in partnership (e.g. cooperating around and with the older person). Actions taken to manage these evaluations were related to working with a plan (e.g. performing day-to-day work and planning for continued treatment) and collaborative problem-solving (e.g. finding a solution, involving the older person, and communicating with colleagues).

**Conclusion:**

Working conditions and cooperation with colleagues, the older persons and their formal or informal caregivers, emerged as important factors related to the medication evaluation. By adjusting their performance to variations in these conditions, healthcare professionals contributed to the resilience of the healthcare system by its capacity to prevent, notice and mitigate medication problems. Based on these findings, we hypothesize that a joint plan for continued treatment could facilitate such resilience, if it articulates what to observe, when to act, who should act and what actions to take in case of deviations from what is expected.

**Supplementary Information:**

The online version contains supplementary material available at 10.1186/s12913-021-06518-w.

## Background

“Medication without harm” is identified as the third global patient safety challenge by the World Health Organization (WHO) [1]. An adverse drug event (ADE) is often defined as harm to a patient resulting from an intervention related to a medication [2, 3]. Older persons are at increased risk for ADEs due to their higher prevalence of frailty, multiple medical conditions and polypharmacy [4]. Unplanned and avoidable visits to primary care, to the emergency department, or admissions to hospital due to ADEs constitute a burden on both patients and the healthcare system [1]. Interventions intended to reduce ADEs, including medication reviews, have attracted interest but have not yielded a convincing impact on relevant endpoints such as mortality or morbidity [5, 6]. Preventable ADEs in ambulatory settings originate predominately at the prescribing and monitoring stages of the medication use process [7]. Therefore, focusing on these stages ought to be useful. While a medication review is seen as a one-off event; an evaluation of medications, which includes monitoring and follow-up, is an iterative process [8]. Although international guidelines highlight the importance of evaluating effectiveness of prescribed medications and adverse effects for safe treatment of multi-morbidity in older persons [9, 10], it remains unclear how such evaluations are used in everyday practice. For this study, we define evaluation as “an assessment of performance against an established set of goals” with the assessment based on monitoring positive or negative responses of a medication on symptoms or clinical tests [11, 12].

Traditionally, in patient safety, it has been important to understand why and how negative events occur (Safety-I) [13]. With today’s knowledge of safety in complex systems, such as healthcare, a new approach to describe and promote safety has emerged, called Safety-II [14]. In Safety-II the emphasis is on understanding and learning from situations more broadly, based on the notion that ideas for improvement can be identified by learning both from situations that have worked well and from situations that have worked poorly. Strategies to increase medication safety could benefit from adapting a Safety-II approach.

Therefore, to identify opportunities to increase safety, it will help to better understand how evaluations of older persons’ medications work in everyday practice. An interview study with older persons about their experiences regarding evaluation of their medications found that they trusted healthcare professionals to perform such evaluations [15]. Many older persons were interested in participating in the evaluation, even if some perceived it as difficult, for instance, due to lack of time to discuss on-going treatment or problems in understanding or remembering information. Healthcare professionals’ perspectives on medication evaluation in older persons remains less clear. In Sweden, as in many countries, it is mainly physicians in primary care who prescribe medications for older persons [16]. If older persons are not able to manage the medications by themselves, or with the help of formal or informal caregivers such as family members or homecare staff, support can be provided from a primary healthcare centre or home healthcare services. Home healthcare services are provided by nurses, generally employed by a municipality, to individuals living in nursing homes or in their own homes. Pharmacists are not yet involved on a regular basis in primary care in Sweden. In some areas in Sweden, primary healthcare centres have appointed care coordinators, typically trained nurses, to support and coordinate care services for patients with a substantial need for support.

Based on this background, the aim of this study was to explore physicians’ and nurses’ experiences from evaluations of older persons’ medications, and their related actions to manage concerns related to the evaluations.

## Methods

### Design

The study was conducted using a qualitative, inductive approach according to Flanagan’s critical incident technique (CIT) [17]. In CIT, a critical incident is understood as a retrospective situation with a beginning and an end, and with a clearly positive or negative consequence [18]. The method is useful for gathering important facts about individuals’ experiences of and actions taken regarding a real and defined phenomenon (here, medication evaluations) [18, 19].

### Setting and recruitment

For this study, we recruited physicians and care coordinator nurses at primary healthcare centres, and municipality-based home healthcare nurses from a county in southern Sweden. The directors of the selected centres signed approval for us to recruit participants among their staff. We sent one general invitation by e-mail to all physicians (149 individuals) and care coordinators (69 individuals) at 34 primary healthcare centres. The area manager in one municipality similarly contacted nurses in home healthcare (248 individuals). In CIT, the number of interviews needed depends on the volume of recorded critical incidents, aiming for more than 100 in total [18, 19]. Among the 41 individuals interested in participating, we sought to achieve variation in terms of profession, gender and geographic area. One of the authors (MH) contacted the selected participants by e-mail to provide further information about the study and confirm their willingness to participate. If they agreed to participate, an interview was scheduled. For participants’ convenience, they were invited to choose the time and place for the interview. Before each interview, participants received both written and oral information about the study and they provided informed written consent. After 29 individual interviews, we deemed to have identified a sufficient number of critical incidents. Demographic characteristics of the participants are presented in Table 1.

**Table 1:** Self-reported demographic characteristics of participants

Occupation	Participants, total (female/ male)	Age (years), median (range)	Number of years in the profession, median (range)	Number of years at the workplace, median (range)
Physicians	13 (5/8)	46 (36–63)	21 (9–48)	10 (1–23)
Care coordinator nurses	9 (9/0)	52 (44–61)	25 (12–40)	12 (1–24)
Municipality based nurses	7 (6/1)	42 (26–58)	10 (3–28)	7 (2–24)
**Healthcare professionals**	**29 (20/9)**	**47 (26–63)**	**19 (3–48)**	**10 (1–24)**

### Data collection

Data were collected from individual semi-structured face-to-face interviews in Swedish. The interview guide used the CIT structure, focusing on medication evaluation [18]. The interviews opened with “Please, describe a situation regarding the evaluation of an older person’s medications, that worked well or not that well”, followed by questions addressing “can you describe the situation?”, “why do you think the situation occurred?”, “what was the consequence of the situation?”, “how was the situation handled?”, “how did you experience the situation?” and “has the situation changed your way of working?” (Supplementary file 1). At the end of each interview, participants were asked if they wanted to add anything of relevance regarding the evaluation of older persons’ medications that had not been addressed during the interview. The guide was pilot tested with three participants, which yielded minor clarifying adjustments. The content of the pilot interviews did not differ from the subsequent interviews and they were, therefore, included in the analysis. One of the authors (MH), a pharmacist working with medication safety, conducted all interviews between June and December 2018. The interviews took place at each of the participants’ workplace. All interviews were audio-recorded and took 29 min on average (range, 16 to 36 min) [18]. The interviews were transcribed verbatim. Data were de-identified to maintain confidentiality and were presented in a way that avoided participant identification to protect their privacy and integrity.

### Data analysis

Before the analysis started, the authors clarified the meaning of experiences and actions. Experiences consisted of what the participants experienced as meaningful related to medication evaluations, whereas actions represented the response they subsequently took. The interviews were read several times to gain familiarity with the content. Real and defined descriptions regarding medication evaluation were identified and included in the analysis [17, 18]. Actions and experiences were analysed separately, starting with critical incidents defined as actions. Each identified action was condensed and coded based on content. Codes with similar content were grouped to form subcategories and then, by abstraction, categories and finally main areas. The same procedure was then done for the experiences (Tables 2 and 3). Two of the authors performed most of the analysis, but the analytical process was discussed within the research group until consensus was reached.

**Table 2:** Summary of healthcare professionals’ experiences of medication evaluation (number of critical incidents = 445)

Critical incidents (quotes)	Subcategories	Categories	Main areas
Sometimes it is the healthcare centre and sometimes it is the hospital that manage different things. // And then he has some skin problems as well, and is connected to Dermatology as well. // Then it becomes very difficult for them to see kind of a whole. [Person13]	Collaboration between colleagues (14 + 25 = 39)	**Co-operation around the older person** (117)	**Working in partnership** (222)
You know, she [*the patient*] has so many contacts with health services overall, so I kind of end up in between all these contacts, you could say. [Person10]	Nurse as a coherent link (14 + 18 = 32)		
The wife was very careful at home as well. She weighed her hubby every day and // and then [*performed*] liquid measurement as well. She kept track of how much he drank each day and so on. [Person5]	Formal or informal caregiver as an intermediary (8 + 22 = 30)		
It is one call away, you can have a dialogue directly and then you can change the medication quite quickly // so that the patient gets help, with pain relief for example then. [Person14]	Forum for interaction (0 + 9 = 9)		
And then this information is to be filtered then // through me, that is // yes, right, to evaluate the treatment when the patient cannot speak for themselves. [Person17]	Difficult to evaluate through others (4 + 3 = 7)		
Who has no, so to speak, home healthcare contact. And she has not wanted to receive homecare. [Person23]	Older persons’ autonomy (10 + 22 = 32)	**The older person as a partner** (105)	
He didn’t understand that he was meant to go and re-fill it; instead, he thought it was a limited treatment period. [Person9]	Older persons’ ability to understand (6 + 23 = 29)		
It is difficult to evaluate these blood pressure medications in her case as well. Because we don’t really know what she is taking or if she is taking anything at all. [Person6]	Older persons’ adherence to therapy (5 + 16 = 21)		
Even if the patient is cognitively lucid, it is not so easy if you write and that becomes only sentences for them… [Person8]	Older persons’ need of understandable information (7 + 7 = 14)		
I cannot keep calling all the time either like that, you know. // But she was cognitively capable of contacting us if needed // and I trust that. So, then I feel, that then it will have to be the patient’s responsibility to get in touch. [Person26]	Expectations on older persons (3 + 6 = 9)		
I observe that the patient has had a very hard time with breathing, it is heavy breathing and one sees it very clearly. It becomes evident when it is time to evaluate the effect of diuretics, for example as in this case. [Person18]	Practical application of knowledge (16 + 13 = 29)	**Clinical knowledge and experience** (72)	**Working conditions** (223)
Anyway, she got the best triple treatment recommended today // She was meant to have that, but she, if you had only looked at her, kind of, then you understand that she, she is an old and frail person. [Person25]	Knowledge about medications for older persons (10 + 5 = 15)		
And then it is difficult to evaluate when someone has been taking something [*a medication*] for a long time then. No, but that’s my theory, I don’t know. [Person19]	Complexity in medication evaluation (13 + 1 = 14)		
It is a bit difficult to know cognitively how, how aware this patient was on the whole. Because he sounds very ‘with it’, but he probably is not, we have realized. [Person2]	Difficult to assess self-care in older persons (5 + 8 = 14)		
Then afterwards she actually reported a [*blood*] pressure that was okay. So, I think I had followed it up, and that we kind of accepted it. Because she had around 140 anyway at the healthcare centre. [Person24]	Frequency decrease over time (16 + 18 = 34)	**Situational conditions** (151)	
Then it’s pulse, saturation, and blood pressure primarily. Leg swelling and the patient’s sense of well-being // and symptoms of hallucinations, psychological symptoms. That’s what I wanted, to get updated on. [Person21]	Facilitate by using a plan (16 + 22 = 32)		
It is a lot of medications and often he can show up every now and again for other small issues, that he has a sore foot or some other small thing. And then you never have enough time to go through everything. [Person24]	Resource to perform in a good way (15 + 14 = 29)		
And what is important, as I see it, is I guess that you do not just carry on…like, renew prescriptions and so on. And then you… yes, then you kind of have to think twice and it is rarely such an emergency, that all pills are finished. [Person28]	Medication management trigger (8 + 14 = 22)		
So she gives feedback to me in the same way, through *electronic messaging* and… sometimes we also speak on the phone of course. But… it is a smooth way to communicate quickly so you don’t need to disturb them in their work. [Person27]	Written communication to colleagues (4 + 10 = 14)		
‘That the patient is discharged from here. She has completed her treatment here. We refer this on to the healthcare centre’ and I don’t know what their routines are. Why or where that request for follow-up goes… no? [Person14]	Familiarity with the work of others (2 + 10 = 12)		
I received feedback from the hospital where the patient was admitted due to dehydration, then we realized that this was such a high dose of *diuretics* after the previous interaction. [Person20]	Adverse event due to absence of evaluation (4 + 4 = 8)		

Number in parenthesis indicates number of critical incidents, for the main area and categories (total number), for Subcategories (number for physicians + number for nurses = total number)

Words in italic indicate that a name has been replaced or an explanation when in parentheses. Backslashes indicates that a word or a phrase has been removed

**Table 3:** Summary of healthcare professionals’ actions taken regarding medication evaluation (number of critical incidents = 208)

Critical incidents (quotes)	Subcategories	Categories	Main areas
Yes, I made my latest real attempt at evaluation related to dementia medications, initiating *Medication*, and then I tried to perform that over the phone with the person’s wife. [Person22]	Monitor efficacy and adverse effects (5 + 20 = 25)	**Perform day-to-day work** (56)	**Work with a plan** (94)
And then I look at the value and adjust or plan based on yes, the need. [Person29]	Adjust treatment to individual needs (11 + 1 = 12)		
Then I feed it back directly to the physician as well. So I naturally set a date and a day when I know the physician is present so we can do it at once. [Person16]	Report monitored result to others (0/11 = 11)		
I have dictated the assessment in the medical record. I have asked to have a copy sent to the staff person responsible in home healthcare. [Person20]	Document what emerged at the follow-up (1 + 7 = 8)		
Yes, but then I usually put it on my schedule. Let’s say she calls today and we agree on ‘Now you take *Medication* for a few days and then you weigh yourself and so on and then I’ll call you again on this date’. [Person10]	Book follow-up appointments (2 + 11 = 13)	**Planning for continued treatment** (38)	
Then I send a message to the district nurse that ‘Can you check on this patient here…. with blood pressure [*readings*] in 2 and 4 days and see how it works’. [Person27]	Give instructions to colleagues (9 + 2 = 11)		
I try to be careful about this nowadays, to note the blood pressure target in the medical record. // both for the patient’s sake, if they read their record themselves and also because we do have nurses who work with hypertension. So, if they see the patient, they can see in the medical record, what we are aiming for, so to speak. [Person24]	Share a plan for continued treatment (4/5 = 9)		
Now when this [*request for*] prescription renewal comes in, he does have other pain medications too, but now it was precisely this *Medication*, then, then yes, then you renew it, without making any bigger deal out of it. [Person22]	Renew prescriptions (3/2 = 5)		
But since I know the woman and know who she is, I call, to personally leave the information to her and get back [*information*] that it [*the medication*] does not work at all anyway because she has a huge stomach ache. [Person16]	Communicate with the older person/ their relative (7 + 16 = 23)	**Involve the older person** (57)	**Collaborative problem-solving** (114)
There we probably also fixed it, so she got a little more control over her medications. [Person6]	Simplify medication management (3 + 16 = 18)		
I, we hand over the medication list and kind of go over it. Because there are quite a few patients who don’t always fully know why they take their medicines. [Person24]	Perform a medication reconciliation (4 + 4 = 8)		
I can check the medication bag, I can ask questions about when to take the next medication dose or so. And then I get correct answers, so that it is a kind of follow-up you can actually carry out. [Person12]	Assess older persons ability to cope (2 + 6 = 8)		
And she thinks she should take it that way, so one’s had to contact the physician and ask, ‘Is this really still relevant?’ [Person14]	Ask for instructions when there is no plan (0/14 = 14)	**Communicate with colleagues** (28)	
Yes, and then she hands it over so the staff. And we talk to the caregiver staff when we’re there too. We obviously do that. But then one says this and another one says that. [Person17]	Discuss continued treatment with colleagues (1/6 = 7)		
But then he accumulated fluid, called the cardiology clinic and said that... Because he goes for follow-up visits there and he may possibly need an earlier appointment than planned. [Person13]	Inform colleagues about detected medication related problems (0/7 = 7)		
And then you notice by chance, when you go to review something else on her medication list, that this medication is still there. That she is still taking it and it hasn’t been de-prescribed. [Person14]	Alert medication related problems (6 + 5 = 11)	**Finding a solution** (29)	
Then we made a short-term plan until tomorrow at least and so. // Tomorrow I will probably have enough time to call, you’ll have to put it on my schedule for tomorrow then. [Person5]	Make a plan when it is missing (3 + 8 = 11)		
No, there was no [*note*]. I found no note on the indication for *Medication*. [Person19]	Search for information about plans (2 + 5 = 7)		

Number in parenthesis indicates number of critical incidents, for the main area and categories (total number), for Subcategories (number for physicians + number for nurses = total number)

Words in italic indicate that a name has been replaced or an explanation when in parentheses. Backslashes indicates that a word or a phrase has been removed

### Ethical considerations

The study was approved by the Regional Ethical Review Board in Linköping, Sweden [Dnr 2018/256–32] and it adhered to the Declaration of Helsinki: participants provided written informed consent and their privacy was protected.

## Results

A total of 653 critical incidents, divided into 445 experiences and 208 actions, were identified. Healthcare professionals’ experiences influencing the medication evaluation for older persons in a positive or negative way formed two main areas: working in partnership and working conditions (Table 2). Actions also formed two main areas: working with a plan and collaborative problem-solving (Table 3).

### Experiences

#### Working in partnership

*Cooperation around the older person* concerns how collaboration between healthcare professionals and caregivers can influence medication evaluation. Good collaboration between different healthcare providers (e.g. between hospital and primary care institutions, or different healthcare professionals) was experienced when the professionals involved shared the same picture of what to do or expect and trusted each other’s abilities. Lack of collaboration caused misunderstandings and unclear division of responsibilities for the evaluation process. Primarily, nurses experienced having homecare staff or relatives participate in the medication evaluation both as an asset and as an obstacle, depending on the caregivers’ presence or lack of involvement, knowledge and/ or commitment. The involvement of nurses, both at the primary healthcare centres and in home healthcare, was perceived as an important part of the evaluations, as a point of contact for the older person and as a key factor to monitor the person’s status between visits with the physician. A communication channel, such as a regular physical meeting or a dedicated number to call, seemed to facilitate communication between healthcare professionals and thereby the evaluation process, as reported mainly by the nurses. Evaluating a medication based on information passed on from another person was experienced as challenging because different people might interpret symptoms differently.

*The older person as a partner concerns* experiences of having older individuals actively involved in the medication evaluation. The healthcare professionals experienced that older persons expressed their wish to be involved in the evaluation to be independent, knowledgeable, and make their own observations and decisions. They also observed that persons sometimes used medications in other ways than prescribed and speculated that this could be due to a lack of understanding, an inability to manage their medications, or their own decisions on other grounds, which can lead healthcare professionals to make decisions for continued treatment on erroneous assumptions. Participants reflected on how communication problems or cognitive impairment may hinder older persons from understanding the intentions behind recommended treatments and plans. Providing both written and verbal information that the older person can understand was considered important to avoid misunderstandings. Moreover, some participants described situations where they expected an older person to manage their treatments on their own, including having responsibilities or being able to give feedback to the healthcare professionals.

#### Working conditions

*Situational conditions* concern healthcare professionals’ experiences of the preconditions for medication evaluations. Participants experienced that careful evaluation occurred closely in time (days to weeks) after a medication adjustment, but then gradually subsided and sometimes ceased completely when the medication seemed to work well for the older person. The evaluation was facilitated by a plan on what to monitor and a clear division of responsibility between the healthcare professionals involved. The absence of such a plan could cause medications to be continued with no one questioning their appropriateness. Regular physician visits made an evaluation more likely, as did having enough resources, primarily time and staff. Healthcare professionals who help older persons manage their medications could trigger evaluations; for instance, home healthcare nurses questioned the rationale for treatment when they were asked to renew prescriptions or refill the dosette for a person. The possibility for written communication between staff, including referrals and text messages within the patients’ record, was important to ensure appropriate continuation of medications. A lack of understanding and knowledge about other units’ routines was experienced as sources for misunderstandings. Not reassessing the risks and benefits of a medication for a particular person, for example, continued anticoagulation, was believed to cause an increased risk of harm.

*Clinical knowledge and experience* concerns the ability of healthcare professionals to perform a proper medication evaluation. Practical application of knowledge was experienced when the healthcare professionals had skills to assess the treatments’ suitability for the individual, to use tools such as a visual analogue scale for pain assessment, or to manage the situation based on clinical experience. Participants described their knowledge about medications for older persons (e.g. being up to date on guidelines and proper medications to use) as essential. Even so, the complexity of treatment with medications for older persons can make it difficult, mainly according to the physicians, to determine if symptoms are side effects or normal aspects of ageing. Participants also experienced difficulty in assessing the persons’ ability to manage their medications and adhere to instructions for continued treatment.

### Actions

#### Working with a plan

*Perform day-to-day work* describes healthcare professionals’ routine work when they know how to do a medication evaluation. Close monitoring by nurses of both desired and adverse effects of treatment for the older person was typically undertaken shortly after changes in treatment. Nurses reported findings from such monitoring to the older person’s physician, daily. Findings from monitoring were documented on a regular basis and colleagues could often read others’ notes, if they shared the same patient record. Actions to adjust treatment according to monitoring feedback were undertaken mainly by physicians.

*Planning for continued treatment* refers to planning evaluations in advance in a structured way. Scheduling follow-up appointments was one of the actions taken by the professionals regarding blood pressure measurements or annual check-up visits at the primary healthcare centre, for example. Physicians gave instructions to the other healthcare providers (e.g. nurses) about what to monitor and how to follow up. Both nurses and physicians shared plans for continued treatment with homecare staff or healthcare colleagues. Nurses were more active in implementing the care plans overall, whereas physicians were more active regarding agreements about medications. The actions to continue treatment included renewing prescriptions, either when a nurse asked a physician to do so or when a physician did so directly; sometimes, these renewals were done with no correlation to a medication evaluation.

#### Collaborative problem solving

*Involve the older person* concerns healthcare professionals’ actions towards the older person. Mostly nurses described speaking with the older persons and family members about continued treatment or test results. Steps to make it easier for the older person to understand and comply with their medications by, for instance, initiating dosette dispensing, was aimed at simplifying management of medications. Another concrete action was undertaking a medication reconciliation together with the older person so that the medication list included only medications relevant for the person. Assessing the older person’s ability to manage their treatment often involved a house call to check how the treatment was carried out at home.

*Communicate with colleagues* concerns active communication between healthcare professionals about how to proceed with the older person’s treatment. This included asking for instructions when information about continued treatment was missing and often concerned older persons seeing several different healthcare providers. Discussing continued treatment with colleagues, both homecare staff and healthcare professionals, was useful to address how to deal with continued treatment. Informing a colleague about problems detected in relation to medications to get help to deal with it in a proper way was done to solve such a situation.

*Finding a solution* describes proactive actions taken by healthcare professionals to sustain proper medication treatment. A medication-related problem often came to light by chance, for example, when finding out that an older person had stopped a treatment due to a misunderstanding. Creating a short-term plan for continued treatment, that is, addressing what will happen over the next couple of days, was done to solve a situation when no plan existed. Searching for information about treatments or plans in the patient record was considered time-consuming but was, nevertheless, performed by participants to inform the next step in the treatment.

## Discussion

In this study, the CIT approach explored physicians’ and nurses’ experiences and actions taken during medication evaluations for older persons. Good cooperation and working conditions emerged as central areas that need to be addressed to make the evaluation process work well. Having a plan made the evaluation easier, but when no plan existed, healthcare professionals tended to develop their own solutions to complete the necessary work and maintain safety. We argue that our findings can be interpreted as expressions of resilience. The theory of resilience adopts a Safety-II approach whereby the system, and individuals therein, adjust their performance before, during, or after disturbances in order to maintain desired levels of performance [20, 21]. Four abilities are identified as necessary for a resilient performance: to respond, monitor, anticipate, and learn [22]. Our findings exemplify these abilities and, thereby, as an example of resilient healthcare, indicate opportunities for making medication treatment for older persons safer [20].

Healthcare professionals experienced that their knowledge about proper medications for older persons and their ability to notice and report medication-related problems were crucial for proper medication evaluation. Physicians may prescribe inappropriate medications for older persons due to limited knowledge about medications that are inappropriate for them, inability to judge the suitability of the treatment, or prescribers’ own bad experiences when discontinuing medications [23]. Healthcare professionals may also underestimate the prevalence of ADEs among older persons, because of concurrent medical conditions, and therefore overlook potential ADEs [24, 25]. The ability to anticipate potential risks and responses are necessary for resilient performance [22]. In their day-to-day work, nurses and physicians monitored and adjusted treatment when they knew what to do and what to expect. Therefore, to sustain patient safety, it is important to promote and maintain clinical knowledge among healthcare professionals in relation to medications for older persons, including learning from their observations and gained experiences.

Our findings show that although evaluations occurred often and according to a structured plan at the beginning of the treatment, they tended to become less frequent over time. Time constraints and a lack of long-term plans for treatment hindered good evaluations. Regular visits and sufficient time and resources are important enablers to minimise inappropriate medications for older persons [26]. A German study addressing general practitioners’ views on long-term medications in an older population found that solving acute problems tends to occupy most of the practitioners’ time, thereby reducing time for regular evaluation of long-term treatment [27]. Because medical conditions in older persons may change rapidly, decreased intensity of monitoring over time can threaten patient safety. To ensure clarity about continued treatment and to enable monitoring and proper actions if the patient’s condition changes, written communication between healthcare professionals was considered crucial [27]. Other studies concerning older persons’ care have identified successful exchange and understanding of information regarding continued treatment between physicians, patients, and other healthcare professionals as critical to promote medication safety [28, 29]. Our results emphasize the need for a structured joint plan for continued medications, for all involved to anticipate the next step and support the procedure under the prevailing circumstances over time.

Moreover, our research revealed the importance of good collaboration between colleagues; close cooperation between different healthcare providers seemed to facilitate medication evaluations. When negative situations occurred, participants reported trying to solve the problem and alerting colleagues, which can be seen as important adaptive behaviours [22]. Nurses often acted as a link between the older persons and their physician, playing a central role in monitoring older persons’ medications. A Swedish study indicated that general practitioners rely on home healthcare nurses to coordinate care and treatment for patients at home [30]. Another study found nurses to have an important role in detecting, assessing and reporting medication-related issues in municipality-based care, if given the right conditions to undertake the evaluation [31]. Our study also indicated that unclear division of responsibility could impair teamwork, which, together with communication, has been identified before as a non-clinical skill important to increase patient safety [32]. Robust, established ways within the healthcare team to identify and communicate changes in an older person’s condition, and to know who is responsible to identify and respond to these changes, seems therefore essential to promote resilience in the system and prevent ADEs.

Experiences regarding the older person as a partner in medication evaluations concerned their adherence to treatment, their ability to understand information and their active involvement. Similar reflections were observed when exploring older persons’ views of medication evaluation [15]. Even if older persons themselves and healthcare professionals agreed on the possibility of shared involvement in the evaluation, there were also concerns. In this study, healthcare professionals sometimes found it hard to assess and predict the older person’s abilities, which, if misjudged, could jeopardize safety. Instruments to assess patients’ capacity to manage medications often focus on dispensing and administering medications, not on the individuals’ skills, knowledge, or ability to alert the physician or pharmacy about treatment-related issues [33]. Healthcare professionals also highlighted older persons’ need for understandable information about continued treatment. In a British report on medication management in older persons, individualized information has been proposed as an important intervention to give them a sense of control over their medications [34]. To enhance resilience in the system, healthcare professionals would benefit from better tools to anticipate, monitor, and respond to the variability in older persons’ conditions, and their capacity and willingness to take an active part in their care and its evaluation. When the older persons and healthcare professionals share responsibility about medication treatment, it is important to address each party’s liabilities and skills from a patient safety perspective.

### Methodological considerations

The CIT method allowed us to learn from situations described by healthcare professionals that have either worked well or poorly [17]. To ensure the trustworthiness of our results, we considered credibility, confirmability, dependability, and transferability throughout the data collection and analysis [35].

The multidisciplinary author group, both women and men with experience in qualitative research, included a pharmacist, a nurse, and two physicians. All had different experiences of the topic, both practical and theoretical, which we believe contributed to the study’s credibility, as pre-understanding when designing and conducting CIT is important [17]. At the same time, researchers’ objectivity is important when considering the confirmability of data. Therefore, during the analysis, peer debriefing was used where different step in the analysis and findings were discussed and reflected upon by the authors and with colleagues, with knowledge in CIT, outside the research group. The discussions were important because one of the weakness of the CIT approach is that no specific analysis method is recommended [36]. We have tried to describe the analysis process thoroughly in the Method section to achieve dependability. Flanagan argued that clearly defining what is to be explored in the study is essential when using the CIT approach [17]. During the data analysis, it emerged that the concept of evaluation was interpreted differently among the healthcare professionals, including evaluation of both practical management and patients’ clinical response to treatment. Through discussions, we concluded that the definition of evaluation used in this study covered all those different views.

A potential challenge with CIT is the subjects’ difficulty in remembering incidents correctly, because the memory tends to fade and lose power over time [37]. To promote the study’s credibility, we asked participants to prepare before the interview by re-calling situations they had experienced that worked well or poorly during medication evaluations. During the interviews, participants found it more difficult to remember specific situations that had worked out well, which may have affected our findings, because participants’ ability to describe both positive and negative situations is an important aspect in CIT [19].

By gathering data from nurses and physicians, we have explored critical incidents from a broader perspective, reflecting on the experiences and actions of healthcare professionals involved in medication evaluation for older persons. The study was conducted in a Swedish context, with healthcare professionals commonly involved in medication evaluation. However, healthcare systems vary between countries and this may affect the *transferability* of the findings.

## Conclusion

Working conditions and cooperation with colleagues, the older persons and their formal or informal caregivers, emerged as important factors related to the medication evaluation. By adjusting their performance to variations in these conditions, healthcare professionals contributed to the resilience of the healthcare system and its capacity to prevent, notice and mitigate medication problems. Based on these findings, we hypothesize that a joint plan for continued treatment could facilitate such resilience, if it articulates what to observe, when to act; who should act and what actions to take in case of deviations from what is expected. Further research is needed to evaluate whether such an approach could actually increase medication safety for older persons.

## Supplementary Information

**Additional file 1.**

## Data Availability

After the study was finished, data is kept secure in the Region Jonkoping County. The data and materials used in the current study is available from the corresponding author on reasonable request but only in accordance with the ethical approval.

## Abbreviations

ADE: Adverse Drug Event
CIT: Critical Incident Technique
WHO: World Health Organization
