# Supplementary material for: Evaluation of older persons’ medications: a critical incident technique study exploring healthcare professionals’ experiences and actions
Source: BMC Health Serv Res. 2021 Jun 7;21:557. doi: 10.1186/s12913-021-06518-w (PMC8182897; doi:10.1186/s12913-021-06518-w)
Supplement: Supplementary file 1 — Additional file 1. [file 12913_2021_6518_MOESM1_ESM.docx]

**Interview-guide for CIT**

**Evaluation of older persons’ medications**

**Introduction**

Since you are involved in medication treatment in older persons, I would like to share your experiences and views on how medications are monitored and evaluated.

The aim of this study is to explore physicians and care coordinator nurses at primary healthcare centres, and municipality-based home healthcare nurses experiences from evaluations of older persons’ medications, and their related actions to manage concerns related to the evaluations. We will try to find factors that enable us to improve patient safety and increase patient and relatives' participation in the medication evaluation.

I will ask you to describe a situation regarding the evaluation of an older person's medications that worked out well or not so well and I will ask additional questions to get the situation as complete and whole as possible.

**Demographic characteristics of the participant**

The participants’ age, sex, number of years in the profession and number of years at the workplace were asked for before the interview started.

**Question-guide**

- Please, describe a situation regarding the evaluation of an older person's medications that worked well or not that well.
- **Additional questions:**
  - Can you describe the situation?
  - Why do you think the situation occurred?
  - What was the consequence of the situation?
  - How was the situation handled?
  - How did you experience the situation?
  - Has the situation changed your way of working?

**Rounding up**

Do you want to add anything of relevance regarding the evaluation of older persons’ medications that had not been addressed during the interview?
